# Intermolecular Interactions in the Polymer Blends Under High-Pressure CO_2_ Studied Using Two-Dimensional Correlation Analysis and Two-Dimensional Disrelation Mapping

**DOI:** 10.1177/0003702820978473

**Published:** 2021-01-07

**Authors:** Huiqiang Lu, Hideyuki Shinzawa, Sergei G. Kazarian

**Affiliations:** 1 Department of Chemical Engineering, Imperial College London, SW7 3AZ, London, UK; 2 Research Institute for Sustainable Chemistry, National Institute of Advanced Industrial Science and Technology (AIST), Ibaraki, Japan

**Keywords:** High-pressure CO_2_, interactions, polymers, in-situ attenuated total reflection Fourier transform infrared spectroscopic imaging, ATR FT-IR, polycaprolactone, poly(lactic acid), two-dimensional correlation analysis, 2D-COS

## Abstract

Exposing polymers to high-pressure and supercritical CO_2_ is a useful approach in polymer processing. Consequently, the mechanisms of polymer–polymer interaction under such conditions are worthy of further investigation. Two-dimensional correlation analysis and two-dimensional disrelation mapping were applied to datasets of polycaprolactone –poly(lactic acid) blend with or without high-pressure CO_2_ obtained using in situ attenuated total reflection Fourier transform spectroscopic imaging. The relatively weak dipole–dipole intermolecular interactions between polymer molecules were visualized through the disrelation maps for the first time. Because of the specially designed polymer interface, the interactions between the same type of polymer molecules and different types of polymer molecules were differentiated. Under exposure to high-pressure CO_2_, all three types of interactions: interaction between polycaprolactone molecules and poly(lactic acid) molecules, interaction between polycaprolactone molecules and interaction between poly(lactic acid) molecules become weaker than those in the polymer interface without high-pressure CO_2_. The resulting increase in the Flory interaction parameter is the main cause of phase separation in the PCL–PLA blend under high-pressure CO_2_. The findings from this study will be of benefit for polymer processing with high-pressure and supercritical CO_2_.

## Introduction

Because of its low toxicity, flammability, and cost, high-pressure and supercritical CO_2_ is becoming more popular in many industrially relevant processes so as to make them “greener”.^1^ In contrast with organic solvents, it does not contaminate the final product as it is trivially removed by reducing the pressure. Moreover, it can reduce the glass-transition temperature of polymers and hence increase their chain and segmental mobility temporarily, which is known as plasticization.^2^ Consequently, as a plasticizing agent, viscosity modifier, reaction medium and foaming agent, high-pressure and supercritical CO_2_ is widely applied in polymer processing, such as extraction, foaming, supercritical fluid dyeing and crystallization.^3,4^

In order to fully realize the potential of polymer processing with high-pressure and supercritical CO_2_, the mechanisms of polymer–polymer interactions require further investigation.^5^ To be more specific, the non-covalent bonds between molecular chains prevent the interaction between additives and functional groups of polymers and then inhibit the access of these additives into the space between polymer molecules. As a result, it has an adverse effect on the penetration of polymer additives which can modify the polymer properties. Moreover, some hydrogen bond interaction in the polymer blends is pharmaceutically important. The hydrogen bond interaction in poly(ethylene glycol) (PEG)–polyvinylpyrrolidone (PVP) blends, which are widely used as transdermal delivery devices, has a great effect on their elastic and adhesive properties.^2^ As a result, investigation of the intermolecular dipole–dipole interaction can provide accurate prediction and subsequent improvement of the polymer properties.

There are numerous methods of spectral analysis of which one of the most popular is two-dimensional (2D) correlation analysis, proposed by Isao Noda in 1993. ^6^ It was developed to analyze perturbation-induced spectral variation.^7,8^ Through using this technique, spectral intensity is plotted as a function of two different spectral variables, such as wavenumber, frequency, or wavelength.^9^ Then the simultaneous or sequential changes of the correlation intensity of spectral variations are derived as synchronous or asynchronous correlations.^7,10^ 2D correlation analysis, which is widely used for data treatment in Fourier transform infrared (FT-IR) measurements, has been proven to be a powerful tool in the analysis of spatially resolved vibrational spectra.^11^ Since the spectral resolution is enhanced by spreading the overlapped bands over the second dimension, this technology can be used to identify the spectral features caused by different kinds of vibration, which are difficult to distinguish in the original data set.^7,9,12^ It can also be used to study intermolecular interactions.^13–15^

As an extension of conventional 2D correlation analysis, disrelation mapping, proposed by Shinzawa et al. in 2017, is a technique to highlight the specific areas where disrelated variation occurs between Wavenumber 1 (ν_1_) and Wavenumber 2 (ν_2_). To be more specific, a convolution filter, which can be viewed as a spatial filter based on the 2D correlation function, was designed to identify the places where an out-of-phase change in the absorbance of two different spectral bands occurs. As a result, its function is to discover the interaction between two molecules and the temporary presence of intermediate species, which is difficult to detect through visual inspection of the conventional FT-IR spectroscopic images. In their study, FT-IR images of PMMA–PEG blends were subjected to disrelation analysis and then disrelation maps were formed. The strong disrelation intensity on the boundary between these two polymers demonstrates the formation of intermolecular hydrogen bonding.^14,16,17^

It is believed that two species of polymers with carbonyl groups exist when they are exposed to high-pressure CO_2_: a free and an interacted species. The interacted polymers with carbonyl groups can also be divided into two species: one forming interactions with CO_2_ and the other one interacting with the carbonyl groups of other polymer molecules.^18,19^ In terms of the PCL–PLA blend exposed to high-pressure CO_2_, the situation is more complex. Since both PCL and PLA have carbonyl groups, the intermolecular dipole–dipole interactions (C=O···C=O) will occur between a PCL molecule and a PLA molecule as well as between the same type of polymer molecules (PCL molecules or PLA molecules). It is difficult to distinguish them because all of these interactions will lead to a different trend of absorbance change compared with the non-interacting carbonyl groups in the polymer and cause cross-peaks in 2D correlation spectra. Thus, in order to distinguish these three types of intermolecular dipole–dipole interactions (C=O···C=O), a specific PCL–PLA interface was prepared in this work.

Phase separation of PCL–PLA blends under high-pressure CO_2_ was visualized through attenuated total reflection (ATR) FT-IR spectroscopic imaging before. The extent of phase separation was found to increase with increasing temperature, CO_2_ pressure, and exposure time.^20^ A study has investigated the mechanisms of possible interactions occurring in polymers under high-pressure CO_2_. It was assumed that the polymer–polymer interactions are partly broken under high-pressure CO_2_.^19^ However, there is no strong spectroscopic evidence for it. Nevertheless, this result has highlighted the importance of understanding this type of interaction in order to predict polymer behavior and improve polymer properties. In this study, two steps were taken to prove it: (1) investigation of the interactions between polymer molecules before exposure to high-pressure CO_2_, and (2) investigation of the interactions between polymer molecules under exposure to high-pressure CO_2_. ATR FT-IR spectroscopic imaging with 2D correlation analysis and 2D disrelation mapping was used to investigate the interaction occurring in the PCL–PLA interfaces before and under exposure to high-pressure CO_2_.

## Materials and Methods

### Materials

**Figure 1.**
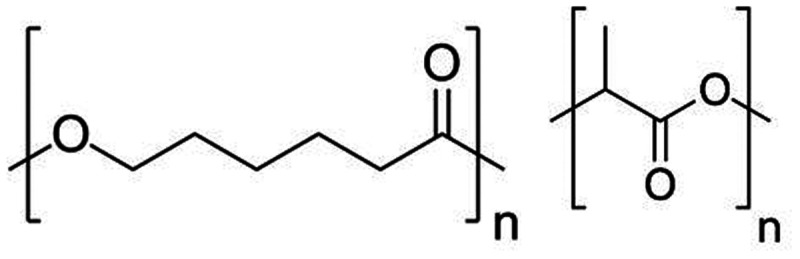
Chemical structures of PCL (left) and PLA (right).

The PCL (Mn = 10 000) was purchased from Sigma-Aldrich. PLA was purchased from Biomer (L9000, MW ≥150 000, D-content = 1.5%). Both of them were used as received without further treatment. The chemical structures of PCL and PLA are shown in Fig. 1. CO_2_ (99.9% pure) was purchased from BOC Industrial Gases (UK).

### Polymer Interface Preparation

The PLA was dissolved in chloroform and then cast onto the diamond to create a PLA film. After the film was dried, half of this film was removed by a scalpel along the central line of the diamond, while the other half was covered by a glass slide. Then a PCL–chloroform solution was placed on the remaining half of the diamond.^21^ Contact between these two polymers was intimate and uniform because PCL–chloroform was liquid when it contacted the PLA film. The size and thickness of PCL half-film and PLA half-film are approximately the same. The ATR FT-IR spectrometer was applied to monitor the concentration of chloroform in the polymer interface. It was confirmed that the chloroform had completely evaporated from the PCL–PLA interface before the measurements were taken.

### In Situ ATR FT-IR Spectroscopic Imaging Measurements

A tensor FT-IR spectrometer (BRUKER Corp.) equipped with a focal plane array (FPA) detector (Santa Barbara Focalplane) was connected with a Golden Gate ATR accessory (Specac) assembled in an IMAC macrochamber. The center of the top surface of the ATR accessory houses a prism shaped diamond ATR crystal. The stainless-steel flat anvil attached to the ATR accessory was used to ensure close contact between the samples and the diamond; 64 × 64-pixel spectroscopic images were recorded simultaneously at a spectral resolution of 4 cm^–1^ on the spectrometer by signal-averaging 64 scans. The obtained data were then analyzed by OPUS (Bruker Corp.) and Matlab. The distribution of the integrated absorbance of a chosen spectral band was plotted to form an ATR FT-IR spectroscopic image. The high-pressure set-up is the same apparatus as that used in previous research from the authors’ research group.^20^

### 2D Correlation Analysis and 2D Disrelation Map

#### Sets of Spatially Resolved Spectra Obtained from Varied Spectral Positions were Processed with 2D Correlation Analysis

Assuming a spectral data matrix A consisting of *m* rows of spectra and *n* columns of spectral variables, a set of dynamic spectra $\overset{∼}{A}$ can be defined by subtracting an average spectrum. The synchronous correlation spectrum Φ is obtained as

(1) $$\Phi =\frac{1}{m-1}{\overset{∼}{A}}^{T}\overset{∼}{A}$$

The absolute value of the disrelation spectrum ${\;}_{\mathrm{ij}}$ is given by

(2) $$|{\;}_{\mathrm{ij}}|=\sqrt{{\;}_{\mathrm{ii}}{\;}_{\mathrm{jj}}-\Phi_{\mathrm{ij}}^{2}}$$

where ${\;}_{\mathrm{ij}}$ means the *i*th row and *j*th column element of the synchronous correlation matrix defined by Eq. 1.

The intensity of a synchronous 2D correlation spectrum ${\;}_{\mathrm{ij}}$ represents the similarity in changes of the spectral intensities at the *i*th and *j*th spectral variables (e.g., wavenumber) observed within a measured area. On the other hand, the intensity of a disrelation spectrum ${\;}_{\mathrm{ij}}$ indicates the dissimilarity of changes of the spectral intensities at the *i*th and *j*th spectral variables appearing in the spatial region.^14,22^ When employing 2D correlation analysis of spectroscopic imaging data, the disrelation intensity plays an especially important role. For example, one can expect to see the emergence of a substantial level of disrelation intensity when chemically or physically meaningful changes occur. This feature of disrelation intensity can be further utilized to construct disrelation maps to identify pertinent spectral variations within a spectroscopic image. In the protocol of disrelation mapping, disrelation analysis is applied to a small select local window within the full spectral image, and the position of the window is then incremented stepwise to cover the entire spectral image. Disrelation intensity thus becomes significant only in the regions where chemically or physically meaningful variations take place. This technique can be used to pinpoint the region of interest within a vast spectral dataset.

## Results and Discussion

### Evidence of Intermolecular Dipole–Dipole Interactions (C=O···C=O) Between Polymer Molecules Before Exposure to High-Pressure CO_2_

The focus of this research is the interfacial region between the PCL bulk area and PLA bulk area. This is because intermolecular dipole–dipole interactions (C=O···C=O) between PCL molecules and PLA molecules can only occur in this region. Moreover, the intermolecular dipole–dipole interactions between PCL molecules only appear in the PCL bulk area, while the intermolecular dipole–dipole interactions between PLA molecules only appear in the PLA bulk area. Thus, these three interactions are well distinguished in the PCL–PLA interface. In order to enhance the intermolecular dipole–dipole interactions between PCL and PLA in the interfacial region, PLA with a higher molecular weight was chosen in this study. The smaller PCL molecules can much more easily enter the free volume of larger PLA molecules, which increases the possibility for PLA to interact with PCL. On the other hand, PLA has a higher proportion of carbonyl groups so that PCL is also more likely to interact with PLA when contacting PLA.

#### ATR FT-IR Spectroscopic Imaging

**Figure 2.**
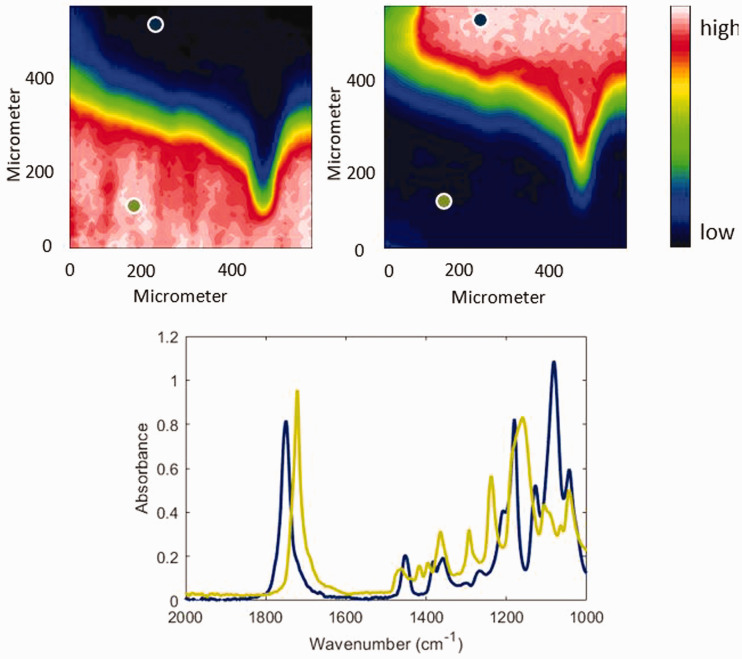
(Top) ATR FT-IR spectroscopic images of the PCL and PLA interface in the absence of CO_2_ at 30℃. The images are based on the spectral band of PCL (1733–1708 cm^–1^) (left) and PLA (1115–1058 cm^–1^) (right). The size of each image is 0.6 mm × 0.55 mm. (Bottom) Spectra were extracted from the PCL bulk area indicated by a brown circle and the PLA bulk area indicated by a blue circle in the top images, respectively.

According to previous research, the band at 1721 cm^–1^ and the band at 1083 cm^–1^ were used to construct the ATR FT-IR spectroscopic images of the distribution of PCL and PLA, respectively.^20^ As shown in Fig. 2, PCL and PLA are fully separated and the interfacial region is clearly indicated by green pixels and blue pixels. It can be observed that the shape of the interfacial area in the center-right of the images is “hook” shaped. The width of the interfacial area is the shortest at the right side of the “hook” and the second shortest at the left side of the hook, which means that the PCL bulk area and the PLA bulk area are much closer in these regions than in other regions of the image.

#### Synchronous Spectra and Disrelation Spectra

**Figure 3.**
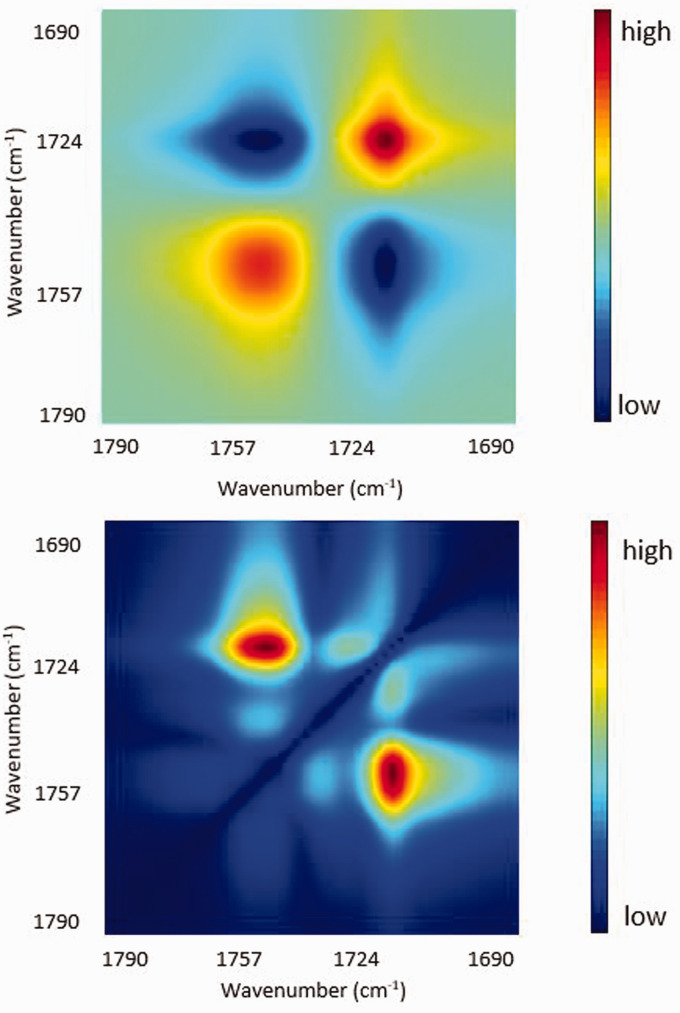
Synchronous (top) and disrelation (bottom) spectra derived from ATR FT-IR spectra of the PCL and PLA interface at 30 ℃.

Synchronous and disrelation spectra derived from the ATR FT-IR spectra of the PCL–PLA interface at 30 ℃ are illustrated in Fig. 3 (top) and (bottom), respectively. As shown in Fig. 3 (top), several auto peaks including (1721 cm^–1^, 1721 cm^–1^) and (1753 cm^–1^, 1753 cm^–1^) can be observed which suggests that the concentration of both PCL and PLA changes to a great extent within this spatial region. This is because the two phases are separated in the polymer interface. The negative synchronous cross peaks including (1721 cm^–1^, 1753 cm^–1^) suggest that the concentration of one polymer increases, while that of the other polymer decreases as a function of spatial position. This also proves the occurrence of phase separation in the polymer interface.

As shown in Fig. 3 (bottom), several cross peaks in the disrelation spectra were observed, which indicates that dissimilar changes to the pattern of spectral absorbance occurred. The cross-peaks (1753 cm^–1^, 1721 cm^–1^) corresponding to the carbonyl group of PLA and that of PCL can also be explained by the phase separation. The auto peaks (1721 cm^–1^, 1721 cm^–1^) and (1753 cm^–1^, 1753 cm^–1^) in the synchronous spectra split into cross peaks (1713 cm^–1^, 1730 cm^–1^) and (1737 cm^–1^, 1751 cm^–1^) in the disrelation spectra. It suggests that the spectral absorbance at 1730 cm^–1^ and 1713 cm^–1^ for carbonyl groups of PCL vary in different manners. Likewise, the spectral absorbance at 1737 cm^–1^ and 1751 cm^–1^ for the carbonyl groups of PLA also vary in different manners. The development of these disrelation correlation peaks reveals the specific states of PCL and PLA molecules in this polymer interface system. It is likely that the changes in absorbance observed at 1713 and 1737 cm^–1^ are ν(C=O) modes of PCL and PLA each representing the unique molecular environment of the components. Such positional shifts of the C=O bands may be explained if one assumes the existence of an intermolecular dipole–dipole interaction (C=O···C=O) between the polymers. The strength of molecular level interactions essentially influences the band position in a vibrational spectrum.^14^ The development of the intermolecular dipole–dipole interaction (C=O···C=O) can appear in the FT-IR spectrum as a band position shift to a lower wavenumber. Consequently, it is proposed that the lower wavenumber bands at 1713 and 1737 cm^–1^ mostly correspond to the interacting carbonyl groups of PCL and PLA, respectively. Although these assignments are not definitive, they are the best current hypothesis to explain the observation.

Figure 3 also shows a cross peak at 1751 and 1760 cm^–1^. It indicates that the origin of the band at 1760 cm^–1^ is different from that at 1751 cm^–1^ (interfacial interaction band). While the origin of this band is not fully understood yet, it is clear that band at 1760 cm^–1^ is not associated with the interfacial interaction band (1751 cm^–1^) and the normal amorphous band of PLA (1737 cm^–1^). Thus, the subtle disrelation peaks at 1751 and 1760 cm^–1^ may represent the presence of a very small amount of crystalline component. Such differences are not readily identified by examination of the original IR spectra. This interpretation of the disrelation spectra demonstrates the power of the 2D correlation technique which can reveal and identify the subtle but important changes in the acquired spectra.

Thus far, it can be concluded that interaction between carbonyl groups occurs. But we cannot determine whether it is intermolecular dipole–dipole interaction (C=O···C=O) between PCL molecules and PLA molecules or between the same type of polymer molecules. As a result, the disrelation map was applied for further investigation.

#### Disrelation Map

As shown in Fig. 2, PCL and PLA diffuse into each other when they contact, and PCL coexists with PLA in the interfacial area. As mentioned above, PCL and PLA are more likely to interact with each other in the interfacial area because of the specific design of the polymer interface. As shown in these ATR FT-IR spectroscopic images (Fig. 2), PCL concentration decreases gradually from the bottom to the top, while PLA concentration decreases gradually from the top to the bottom. The narrower the interfacial area, the higher the concentration of PLA and PCL are on both sides of the interfacial area. As a result, the intermolecular dipole–dipole interactions (C=O···C=O) between PCL molecules and PLA molecules are more likely to occur in the narrower interfacial region.

A convolution filter which can be viewed as a spatial filter based on the 2D correlation function was applied to highlight the areas where disrelated variation between ν_1_ and ν_2_ occurs.^14^ It can highlight the intermolecular dipole–dipole interactions (C=O···C=O) between polymer molecules. The smallest size of convolution window: 3 × 3 was used to enhance the contribution from all correlation intensity.

**Figure 4.**
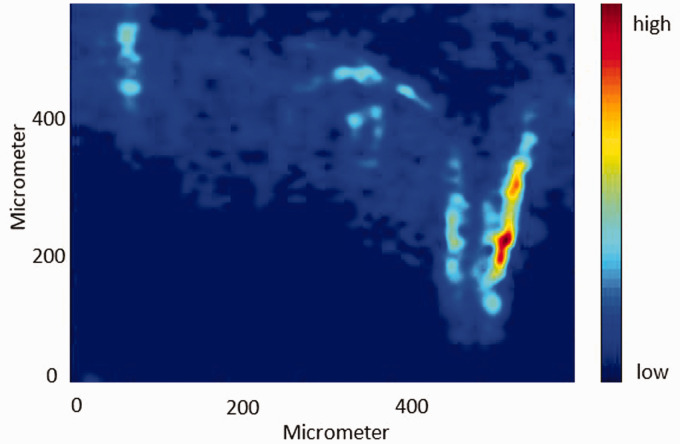
Disrelation map of the PCL–PLA interface obtained by calculating disrelation intensity between 1751 cm^–1^ and 1737 cm^–1^, corresponding to the non-interacting carbonyl groups and interacting carbonyl groups of PLA, respectively.

In order to visualize the different trends of the change in the spectral absorbance of interacting carbonyl groups and non-interacting carbonyl groups of PLA, the disrelation map derived from the spectral absorbance of the bands at 1751 cm^–1^ and 1737 cm^–1^ is shown in Fig. 4. It can be found that the appearance of disrelation intensity indicated by the bright blue is mainly in the same area as the interfacial region and PLA bulk area in the ATR FT-IR spectroscopic images (Fig. 2). The appearance of disrelation intensity in these areas suggests that there is an additional contribution accelerating or delaying the absorbance variation of the band of the carbonyl groups in PLA. In other words, the absorbance change in the band of the interacting carbonyl group of PLA does not follow the trend of that of the non-interacting carbonyl group of PLA. It indicates the appearance of interaction between PLA molecules in the interfacial region and PLA bulk area.

If the only interaction that exists is between the same type of polymer molecules, the disrelation intensity in the interfacial area will be weaker than that in the PLA bulk area because the PLA concentration in the interfacial area is less than that in the PLA bulk area. However, it can be found that the disrelation intensity in the interfacial area is much stronger than that in the PLA bulk area. It suggests that there is additional contribution accelerating or delaying the absorbance variation of band of the carbonyl groups of PLA and it only occurs when PLA contacts PCL. In addition, the disrelation intensity becomes stronger with decreasing width of the interfacial area and peaks at the right side of the hook shape interfacial area which is the narrowest interfacial region. It means that this additional contribution becomes stronger with increasing concentration of contacting PLA and PCL. As a result, the interaction between the PLA molecules and the PCL molecules is also proven to exist.

**Figure 5.**
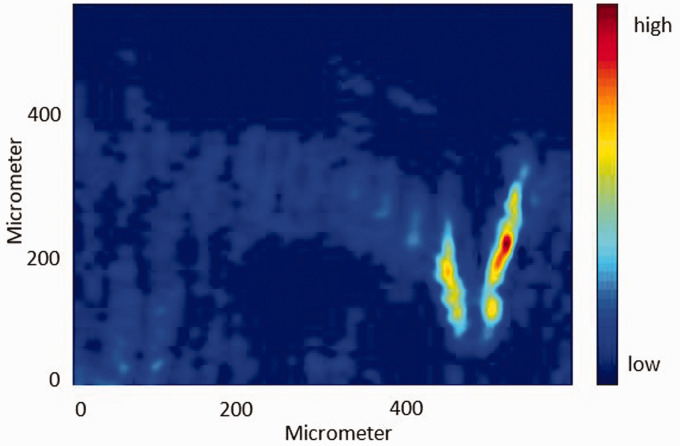
Disrelation map of the PCL–PLA interface obtained by calculating disrelation intensity between 1730 cm^–1^ and 1713 cm^–1^, corresponding to the non-interacting carbonyl groups and interacting carbonyl groups of PCL, respectively.

Likewise, in order to visualize the different trends of the absorbance change in the bands of interacting carbonyl groups and non-interacting carbonyl groups of PCL, a disrelation map derived from the spectral absorbance of the bands at 1730 cm^–1^ and 1713 cm^–1^ is shown in Fig. 5. Likewise, the appearance of disrelation intensity in the PCL bulk area indicates the existence of the interaction between PCL molecules. However, the strength of interaction is much weaker than that of the interaction between PLA molecules. Since the proportion of carbonyl groups in PCL is lower than that in PLA, fewer dipole–dipole interactions (C=O=C=O) will occur in PCL with the same weight as PLA.

The appearance of disrelation intensity in the interfacial area also indicates the interaction of carbonyl groups in PCL with carbonyl groups in PLA. Likewise, the disrelation intensity becomes stronger with the decreasing width of the interfacial area and peaks at the right side of the hook shape interfacial area which is the narrowest interfacial region. It means that this additional contribution, which can accelerate or delay the absorbance variation of the band of the carbonyl groups of PCL, becomes stronger with increasing concentration of contacting PLA and PCL. This result provides more evidence of the occurrence of the interaction between PCL molecules and PLA molecules.

To sum up, the appearance of disrelation intensity in the PCL bulk area indicates the interaction between PCL molecules, while the appearance of disrelation intensity in the PLA bulk area indicates the interaction between PLA molecules. The strong disrelation intensity in the interfacial area of PCL and PLA indicates the interaction between the PCL molecules and the PLA molecules. In fact, a previous study based on ATR FT-IR spectroscopy also provides evidence of such development of intermolecular dipole–dipole interactions (C=OC=O).^18^

### Evidence of Intermolecular Dipole–Dipole Interactions (C=O···C=O) Between Polymer Molecules Under Exposure to High-Pressure CO_2_

#### ATR FT-IR Spectroscopic Images

**Figure 6.**
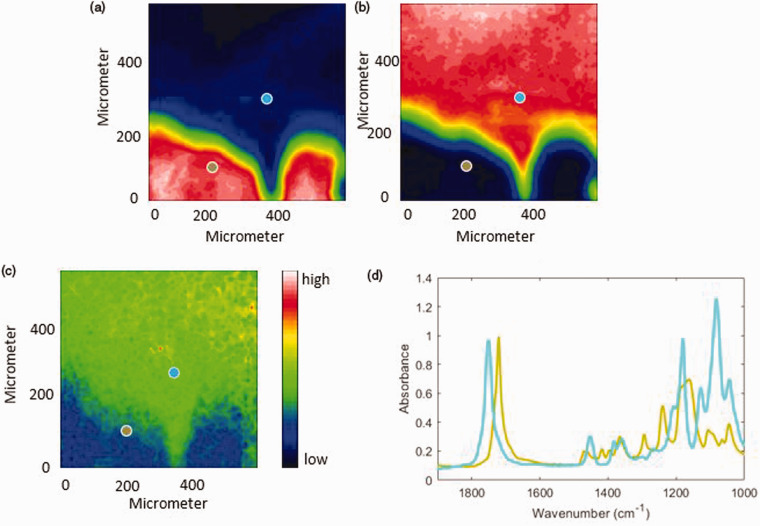
(Top) ATR FT-IR spectroscopic images of the PCL and PLA interface with 30 bar CO_2_ at 30 ℃. The images are based on the spectral band of PCL (1733–1708 cm^–1^) (a), PLA (1115–1058 cm^–1^) (b) and CO_2_ (2355–2315 cm^–1^) (c). The size of each image is 0.6 mm × 0.55 mm. (Bottom) Spectra were extracted from the PCL bulk area indicated by a brown circle and the PLA bulk area indicated by a blue circle in the images, respectively.

As shown in Fig. 6, phase separation still occurs in the PCL–PLA interface under exposure to 30 bar CO_2_ but the interfacial area is closer to the bottom side of ATR FT-IR spectroscopic images. From Fig. 6c, it can be found that PLA absorbs more CO_2_ than PCL, which may be because PLA has a higher proportion of carbonyl groups for the Lewis acid–base interaction and then results in a greater affinity for CO_2_. As the CO_2_ sorption results in the polymer swelling, the more CO_2_ absorbed, the greater the extent of swelling of the polymers. Consequently, the greater extent of PLA swelling leads to displacement of the interfacial area. In addition, a new PCL–PLA interfacial region appears in the bottom right side of the ATR FT-IR spectroscopic images. According to our previous research, peak shifts of the C=O stretching band will occur if crystallization is induced.^23^ In addition, even CO_2_ at 60 bar cannot induce crystallization of PCL and PLA in PCL–PLA blends.^20^ There is no strong spectroscopic evidence of crystallization, such as peak shift, in the spectra shown in Fig. 2, (bottom) and Fig. 6 (bottom), so it is suggested that no crystallization occurs at the interface under high-pressure CO_2_. Importantly, we assume that the intermolecular interactions studied in this work, occur between molecules of different polymers in the amorphous interfacial region because the molecules in the crystalline domains are already involved in interactions within corresponding crystalline domains.

#### Disrelation Map

**Figure 7.**
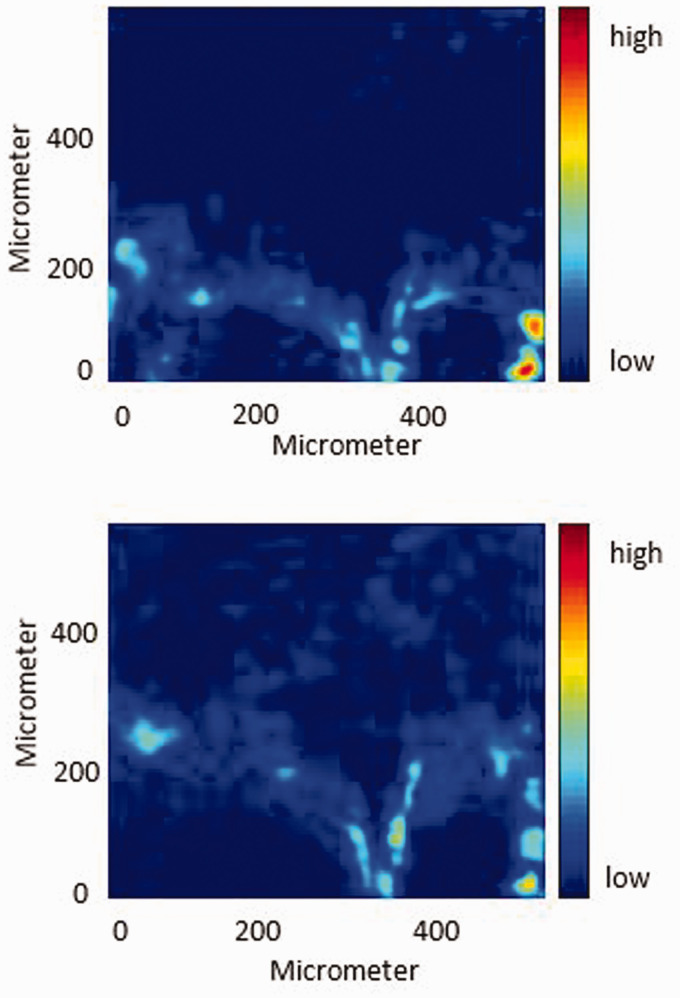
Disrelation maps of the PCL–PLA interface under exposure to 30 bar CO_2_ obtained by calculating disrelation intensity between 1730 cm^–1^ and 1713 cm^–1^ (top), corresponding to the non-interacting carbonyl groups and interacting carbonyl groups of PCL, respectively, as well as disrelation intensity between 1751 cm^–1^ and 1737 cm^–1^ (bottom), corresponding to the non-interacting carbonyl groups and interacting carbonyl groups of PLA, respectively.

The disrelation map derived from the spectral absorbance of the bands at 1713 cm^–1^ and 1730 cm^–1^ (interacting carbonyl groups and non-interacting carbonyl groups of PCL) is shown in Fig. 7 (left). Likewise, the disrelation map derived from the spectral absorbance of the bands at 1737 cm^–1^ and 1751 cm^–1^ (interacting carbonyl groups and non-interacting carbonyl groups of PLA) is shown in Fig. 7 (right). It is found that the disrelation intensity in the PCL bulk area and that in the PLA bulk area are both weaker under exposure to high-pressure CO_2_. It can be concluded that high-pressure CO_2_ breaks some of the interaction of PCL molecules and part of the interaction of PLA molecules. Meanwhile, it is observed that the disrelation intensity shown by the bright blue is in the same area as the interfacial region in the ATR FT-IR spectroscopic images (Fig. 6). The disrelation intensity becomes stronger with the decreasing width of the interfacial area and peaks at the right side of the hook shape interfacial area which is the narrowest interfacial region. It suggests that the interaction of PCL molecules and PLA molecules still exists. However, the strength of the disrelation intensity is much weaker, which means that high-pressure CO_2_ breaks part of the intermolecular dipole–dipole interactions (C=O···C=O) between PCL molecules and PLA molecules. The abnormally strong disrelation intensity in the bottom right side of the disrelation map is caused by the fact that this interfacial area is just formed under exposure to high-pressure CO_2_. Consequently, high-pressure CO_2_ has less time to penetrate the newly formed interfacial area than the former interfacial area.

### The Δχ effect on phase separation of polymer blend under exposure to high-pressure CO_2_

Previous studies based on ATR FT-IR spectroscopy provided evidence of the development of C=O···CO_2_ complex structures.^18,24,25^ After CO_2_ penetrates into the PCL–PLA blend, the Lewis acid–base interaction between CO_2_ and these two polymers will occur. The obtained results can explain the occurrence of phase separation in this PCL–PLA blend under high-pressure CO_2_ observed through ATR FT-IR spectroscopic imaging.^20^ From a thermodynamic standpoint, the Gibbs free energy (free enthalpy G) calculated by Eq. 1 should be positive if phase separation occurs.

(3) $$\frac{\Delta {\text{G}}_{\text{m}}}{\text{RTV}}=\frac{{\;}_{1}}{{\text{N}}_{1}}\text{In}{\;}_{1}+\frac{{\;}_{2}}{{\text{N}}_{2}}\text{In}{\;}_{2}+{\;}_{1}{\;}_{2}\chi_{12}$$

where ΔG_m_ is the free energy of mixing, T is the temperature, R is the gas constant, V is the volume of system, N_1_ and N_2_ are the molar volumes of PCL and PLA, respectively. φ_1_ and φ_2_ are the volume fractions of PCL and PLA, respectively. χ_12_ is the Flory interaction parameter.

The CO_2_-weakened specific intermolecular interaction between the polymers is proven by Figs. 4, 5, and 7, while the appearance of Lewis acid–base interaction between carbonyl groups and CO_2_ is shown in the literature.^18,24,25^ Since the sum of $\chi_{\text{PCL},{\text{co}}_{2}}$ and $\chi_{\text{PLA},{\text{co}}_{2}}$ is larger than $\chi_{\text{PCL},\text{PLA}}$, χ increases and the phase boundary in phase diagram shifts to lower temperatures.^20,26^

## Conclusion

In this work, 2D correlation analysis and 2D disrelation mapping were used to investigate the mechanisms of polymer–polymer interactions. A specially designed PCL–PLA interface was prepared in order to distinguish the intermolecular dipole–dipole interaction (C=O···C=O) between PCL molecules and PLA molecules from that interaction between the same type of polymer molecules. The synchronous and disrelation spectra derived from the ATR FT-IR spectra of the PCL–PLA interface indicate the appearance of interactions between carbonyl groups. Visual inspection of the disrelation map reveals the spatial distribution of these three interactions: the interaction between PCL molecules mainly in the PCL bulk area, the interaction between PLA molecules mainly in the PLA bulk area, and the interaction between PCL molecules and PLA molecules in the interfacial area. Consequently, it suggests that all of these interactions occur in the PCL–PLA interface. Under exposure to high-pressure CO_2_, visual inspection of the disrelation map reveals that these three types of intermolecular dipole–dipole interactions (C=O···C=O) between polymer molecules are weaker. Thus, the 2D correlation analysis provides important information about the break of some of the existing polymer–polymer interactions. The intermolecular dipole–dipole interaction between polymer molecules inhibits the access of polymer additives which can modify the polymer properties. As a result, this investigation of breaking intermolecular dipole–dipole interactions by high-pressure CO_2_ is beneficial for the improvement of polymer properties. The CO_2_-weakened specific intermolecular interaction between polymers and the appearance of Lewis acid–base interactions between carbonyl groups and CO_2_ lead to an increase of the Flory interaction parameter and then shift the phase boundary in the phase diagram to lower temperatures, which is the main cause of phase separation in this blend under high-pressure CO_2_.

Finally, it demonstrates the power of combining FT-IR imaging with 2D correlation analysis and 2D disrelation mapping to identify different molecular interactions. The approach is not limited to the polymer interface with carbonyl groups and has great potential to study other homopolymers or multicomponent polymer systems with different functional groups. This study is the first step toward the realization of this potential.
